# Rare Case of Undifferentiated Embryonal Sarcoma of the Liver that Contained an Adenocarcinoma Component

**DOI:** 10.70352/scrj.cr.25-0200

**Published:** 2025-07-29

**Authors:** Akira Watanabe, Yasuaki Enokida, Masaki Suzuki, Takahiro Takada, Tomonori Yoshida, Jun Imaizumi, Makoto Sohda, Misa Iijima, Hitoshi Ojima, Ken Shirabe

**Affiliations:** 1 Department of Gastroenterological Surgery, Gunma Prefectural Cancer Center, Ohta, Gunma, Japan; 2 Department of Pathology, Gunma Prefectural Cancer Center, Ohta, Gunma, Japan; 3 Department of General Surgical Science, Division of Hepatobiliary and Pancreatic Surgery, Graduate School of Medicine, Gunma University, Maebashi, Gunma, Japan

**Keywords:** adenocarcinoma, carcinosarcoma, liver, neoplasms, germ cell, embryonal

## Abstract

**INTRODUCTION:**

Undifferentiated embryonal sarcoma (UES) of the liver (UESL) is a tumor that is rare in adults. UESL is composed of undifferentiated mesenchymal tumor cells, with characteristic normal bile duct cells found at the tumor periphery. We present a rare case of UESL containing an adenocarcinoma component in one part of the tumor.

**CASE PRESENTATION:**

A 68-year-old male had a tumor with an 8-cm diameter in the left lateral liver segment. CT showed a cystic tumor with a solid component and a heterogeneous area. In the solid component, the contrast effect was weak in the arterial phase and uniform in the late phase. Imaging suggested that the internal tumor area was necrotic or hemorrhagic. We diagnosed it as hemorrhagic hepatocellular carcinoma or combined hepatocellular–cholangiocellular carcinoma. The patient exhibited normal liver function. Left lateral segmentectomy was performed. Macroscopically, the tumor was cystic, with hemorrhagic/necrotic deposits and a solid portion composed of tumor cells. Histopathologically, the tumor had 2 components. One was a nonepithelial tumor comprising immature spindle-shaped and stellate plaques, with positivity for the mesenchymal markers vimentin and CD68 and negativity for myogenic, neurogenic, and other markers. This component was diagnosed as UES. The other component comprised epithelial and adenocarcinoma tissues with differentiated bile duct cells. The tumor was mainly composed of UES cells, with the adenocarcinoma component located predominantly in the periphery.

**CONCLUSIONS:**

We present a unique case of UESL containing UES cells and adenocarcinoma cells at the periphery. A combination of UESL and adenocarcinoma is extremely rare, even among hepatic carcinosarcomas.

## Abbreviations

FDG-PET
: ^18^F-fluorodeoxyglucose positron-emission tomography
HCS
: hepatic carcinosarcoma
UES
: undifferentiated embryonal sarcoma
UESL
: undifferentiated embryonal sarcoma of the liver

## INTRODUCTION

UESL was first described by Stocker and Ishak in 1978.^1)^ Fifty percent of UESLs occur in children aged 6–10 years, and UESL ranks second only to hepatoblastoma and hepatocellular carcinoma in pediatric liver tumors.^1)^ However, the incidence of UESL in adults is low. In 2022, Shimagaki et al. reported that approximately 60 adult cases of UESL had been identified to date.^2)^ The clinical symptoms of UESL are nonspecific and include fever, anorexia, and abdominal pain. The tumor is often a large cystic mass, with an average diameter of approximately 14 cm,^3)^ composed of substantial solid components, as well as internal hemorrhagic and necrotic regions.^4)^ Pathologically, it is bordered by a pseudocapsule and is composed of undifferentiated stellate or spindle-shaped mesenchymal cells.^5,6)^ Additionally, normal hepatocytes or bile duct cells surrounding the sarcomatous tumor cells are frequently found at the periphery of the tumor and are considered a characteristic pathological finding of UESL.^1,5,7,8)^

Liver tumors that combine sarcoma and carcinoma within the same tumor are classified as HCS; however, very few cases of HCS composed of UES as the sarcomatous part have been reported.

We here present a rare case of UESL in which adenocarcinoma cells, rather than normal bile duct cells, were focally found in the peripheral area of the UES, unlike previously reported UESLs.

## CASE PRESENTATION

A 68-year-old male underwent abdominal CT to investigate the cause of fever. CT detected an 8-cm diameter tumor in the left lateral segment of the liver. The patient was referred to our department for detailed examination and treatment.

The patient had a history of hypertension, emphysema, and hepatitis C, which had demonstrated a virological response to antiviral drugs 10 years earlier. The patient had no relevant clinical or physical findings.

In terms of tumor markers, his carcinoembryonic antigen (2.5 ng/mL [normal range, <5.0 ng/mL]), carbohydrate antigen 19-9 (8.5 U/mL [normal range, <37 U/mL]), and protein induced by vitamin K absence or antagonist-II (19 mAU/mL [normal range, <40 mAU/mL]) were within the normal ranges. On the other hand, α-fetoprotein (42.7 ng/mL [normal range, <20 ng/mL]) and α-fetoprotein L3 fraction (93.8% [normal range, <10%]) levels exceeded the normal range.

**Fig. 1:**
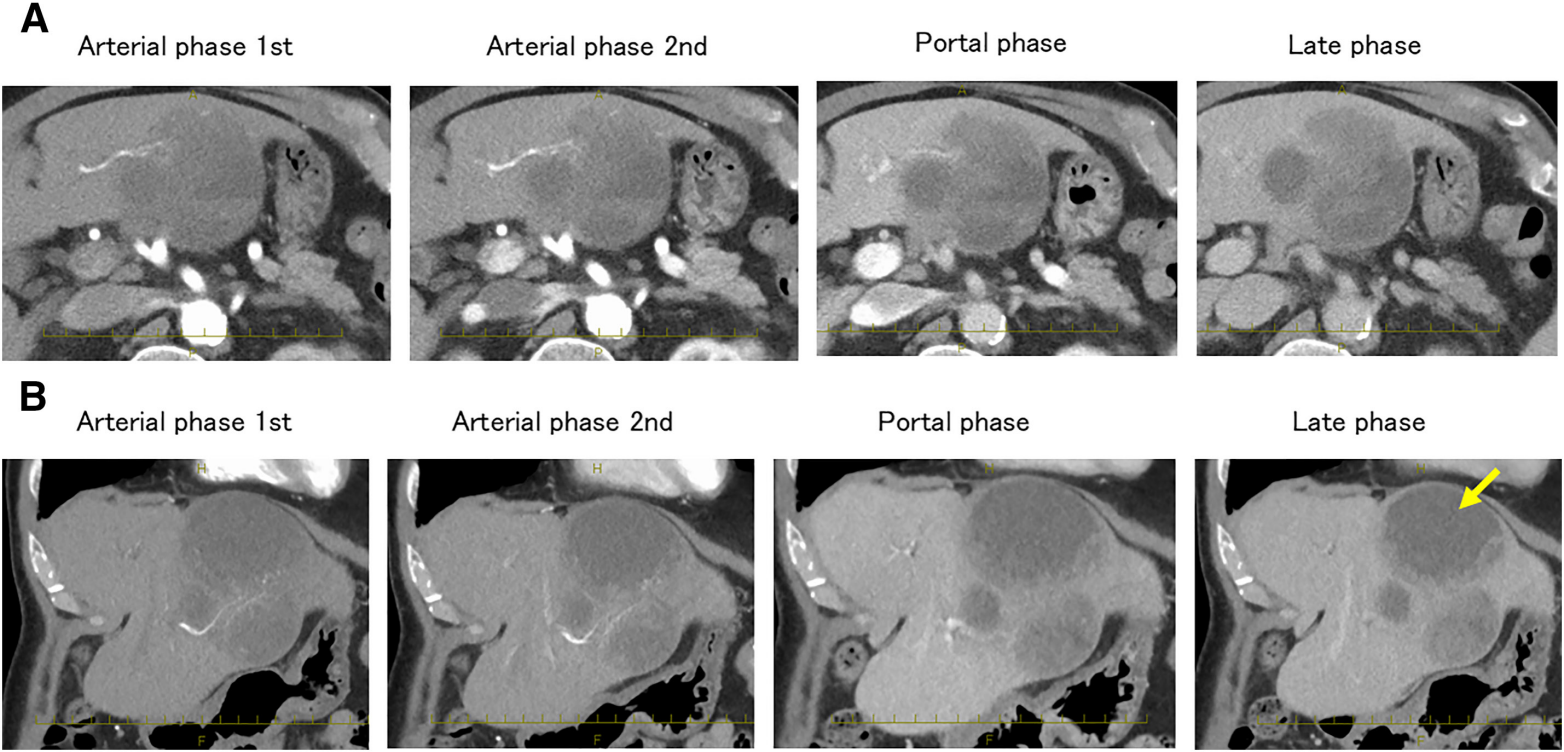
Abdominal CT findings for the tumor (**A**). Panel (**A**) shows axial 4-phase dynamic contrast-enhanced CT images of the tumor. The solid component shows contrast effects during the first and second arterial phases. In the portal and delayed phases, the contrast effect is spread over a relatively large area within the tumor. The border of the tumor is relatively clear (**B**). Panel (**B**) shows coronal CT images of the tumor. A solid tumor component is seen on the foot side of the tumor, while a liquid component (yellow arrow) is observed on the head side.

**Fig. 2:**
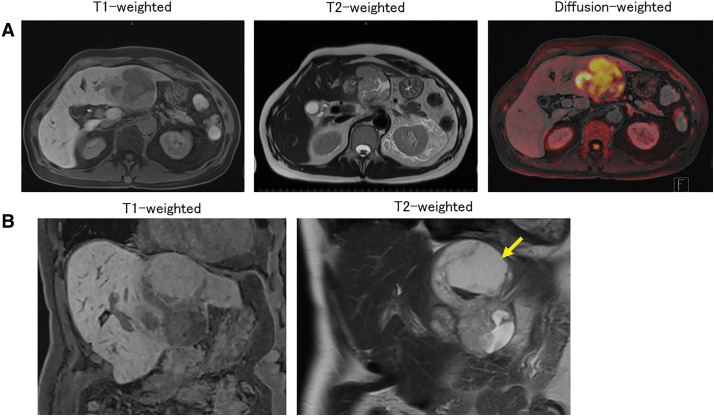
MRI findings for the tumor (**A**). Panel (**A**) shows axial magnetic resonance images of the tumor, which shows a low signal on the T1-weighted image, primarily a low signal along with a high signal area that appears to represent liquid on the T2-weighted image, and a high signal on the diffusion-weighted image (**B**). Panel (**B**) shows coronal magnetic resonance images of the tumor, showing a cystic and liquid component (yellow arrow) on the head side.

**Fig. 3:**
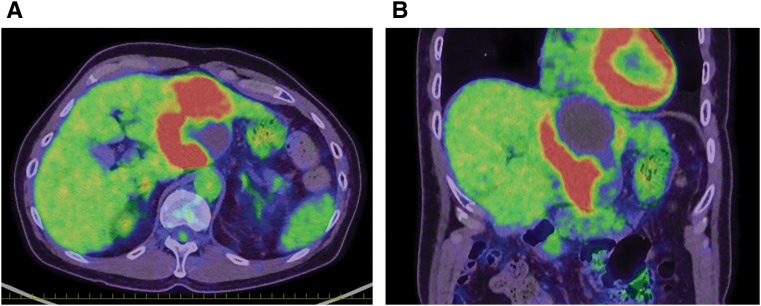
FDG PET findings for the tumor (**A**). Panel (**A**) shows axial FDG PET images of the tumor. High FDG uptake (SUVmax 15.96) is observed in the solid area. The tumor area without FDG uptake is suspected to be necrotic or hemorrhagic on the basis of imaging findings. No distant metastases are seen (**B**). Panel (**B**) shows coronal FDG PET images of the tumor. FDG, ^18^F-fluorodeoxyglucose

CT revealed a cystic tumor with a solid component as well as a heterogeneous area. In the solid region, the contrast effect was weak in the arterial phase and uniform in the late phase (**Fig. 1**). MRI showed a high diffusion-weighted imaging signal in the marginally enhanced area (**Fig. 2**). FDG-PET revealed FDG uptake (SUVmax 15.96) in the solid component of the tumor. No distant metastases were detected. The internal area of the tumor was suspected to be necrotic or hemorrhagic based on imaging findings (**Fig. 3**). Accordingly, we diagnosed the patient as having hepatocellular carcinoma with hemorrhage or combined hepatocellular–cholangiocellular carcinoma.

The tumor was mainly located in segments 2 and 3 of the liver and did not invade the umbilical portion or root of the left hepatic vein. The patient exhibited normal liver function (total bilirubin, 0.48 mg/dL; ICG-R15, 7.0%). Therefore, curative resection was considered possible. We decided to perform left lateral segmentectomy, as the patient would retain sufficient residual liver volume (73.3%) after the procedure.

During surgery, the tumor was observed in the left lateral segment, particularly in S2, and it compressed Glisson’s branch of segment 2 and the left hepatic vein. There was no invasion of the portal umbilicus, and left lateral sectionectomy was performed as planned. Open surgery was performed, with a surgical duration of 235 min and an estimated intraoperative blood loss of 181 mL. The postoperative course was uneventful, and the patient was discharged 8 days after surgery.

**Fig. 4:**
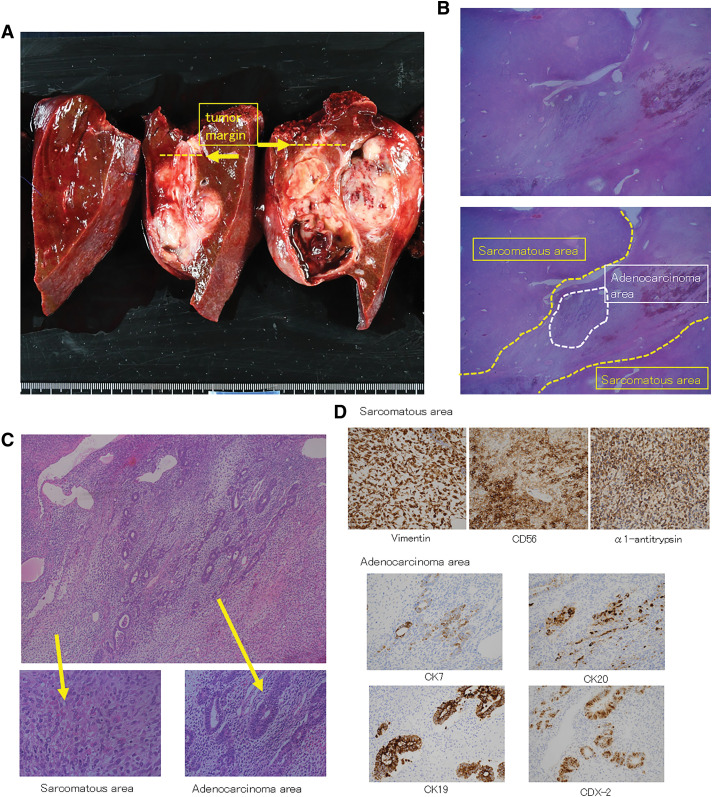
Macroscopic, histopathological, and immunohistochemical findings for the tumor. (**A**) Macroscopic findings for the resected specimen. The tumor primarily comprises a solid component and a partially cystic component containing hemorrhagic or necrotic deposits in the left lateral segment. The tumor margin is indicated by the yellow dashed line, and the white area near the liver resection margin is a fibrotic Glisson’s sheath. (**B**) At low power (hematoxylin–eosin staining, ×15), each component (sarcomatous or adenocarcinoma) is shown by dashed lines. Most of the tumor is occupied by the sarcomatous component (undifferentiated embryonal sarcoma). (**C**) The tumor is mainly composed of undifferentiated sarcomatous cells, with some areas of adenocarcinoma cells thought to have originated from bile duct cells (hematoxylin–eosin staining, ×100). The sarcomatous component includes a nonepithelial tumor with immature spindle-shaped and stellate plaques. These sarcomatous cells show positivity for mesenchymal markers and negativity for myogenic, neurogenic, and other markers. The final diagnosis is undifferentiated embryonal sarcoma. The adenocarcinoma component consists of an epithelial tumor and adenocarcinoma tissue with differentiated bile duct cells (hematoxylin–eosin staining, ×400). (**D**) Findings of immunohistochemical staining. The sarcomatous component shows positivity for mesenchymal markers (vimentin and CD68) and α-1 antitrypsin. The adenocarcinoma component shows positivity for CK7, CK20, CK19, and CDX-2 (hematoxylin–eosin staining, ×100).

The tumor was macroscopically cystic, composed of hemorrhagic/necrotic deposits, and the solid portion was composed of tumor cells (**Fig. 4A**). Microscopic pathological findings revealed that the tumor was composed of 2 types of tumor cells (**Fig. 4B**, **4C**). The first was a non-epithelial tumor consisting of immature spindle-shaped and stellate plaques (**Fig. 4C**) that were positive for α-1 antitrypsin and the mesenchymal markers vimentin and CD68 but negative for myogenic, neurogenic, and other markers. This portion was diagnosed as an UES and accounted for the largest part of the tumor. The second component comprised epithelial tumor and adenocarcinoma tissues along with differentiated bile duct cells (**Fig. 4C**). The adenocarcinoma cells were localized to the peripheral area of the tumor. The adenocarcinoma component showed positivity for the following epithelial markers: CK7, CK20, CK19, and CDX-2 (**Fig. 4D**). **Table 1** shows the results of all immunohistochemical examinations. On the basis of these findings, we diagnosed the tumor as UES with an adenocarcinoma component.

**Table 1:** Immunohistochemistry data of the tumor

	UESL area		Adenocarcinoma area	
Cytokeratin AE1/3	–		+	
Hepatocyte	–		+	Spotted
CEA	–		+	
AFP	–		+	Small number
CD10	+		–	
SMA	–		–	
Desmin	–		+	Spotted
S-100	+	Focal	–	
CK7	–		+	Focal
CK20	–		+	Focal
CK19	–		+	Focal
CDX-2	–		+	
CD56	+		+	Weak
CD34	–		–	
CD117	–		+	Weak
DOG1	–		–	
p53	+		+	
MUC-2	–		+	Small number
Muc-5AC	–		+	Small number
Vimentin	+		+	Focal
CD68	+		–	
α1-Antitrypsin	+		–	
α1-Antichymotrypsin	+		–	
MIB-1-positive rate	90%		90%	

AFP, α-fetoprotein; CEA, carcinoembryonic antigen; SMA, α-smooth muscle actin; UESL, undifferentiated embryonal sarcoma of the liver

After resection, the patient underwent enhanced CT every 3 months and experienced no recurrence during the 1 year following the hepatectomy.

## DISCUSSION

We encountered a rare case of UESL with a focal component of adenocarcinoma within sarcomatous cells. We reviewed and analyzed the clinical, imaging, and pathological characteristics of the tumor. UESL is a liver sarcoma composed of undifferentiated mesenchymal cells.^1)^ Findings of normal liver and bile duct tissue engulfed by sarcomatous cells have been reported previously. However, in this case, the UES was surrounded by adenocarcinoma cells rather than by normal bile duct cells, which is extremely rare and differs from previously reported UESL cases.

UESL was reported to have a poor prognosis in a 1978 study by Stocker and Ishak.^1)^ In a recent report, an analysis of the National Cancer Database in the United States examined the prognosis of 123 cases of UESL (82 children and 41 adults).^3)^ Prognosis is reportedly worse in adult than in pediatric cases of UESL (5-year survival rate: 84.4% in children vs. 48.2% in adults). In pediatric patients, surgical resection and multimodal treatment with adjuvant chemotherapy prolong survival. However, in adult UESL, the lack of established treatment strategies and the differences in tumor biology from that of pediatric UESL may play a role in the poor prognosis. The analysis based on data from the National Cancer Database of the United States indicated that surgical margin positivity after resection, but not tumor size, was a prognostic factor in multivariate analysis.

Shimagaki et al. reported that genetic analysis using FoundationOne companion diagnostics identified 11 somatic mutations, including those in *TP53* and *STK11*, in UESL.^2)^ Loss of function due to *TP53* mutations has been reported in other UESL gene analyses, indicating that p53 may be a useful therapeutic target in the future.^9,10)^ In the present case, immunostaining showed that both the UESL and adenocarcinoma components were p53-positive.

Gabor et al. reported imaging features of 15 cases of UESL.^11)^ On CT, the tumor is often depicted as a well-defined, hypoattenuated cystic lesion. MRI often shows a tumor with a T1-low signal and a liquid component with a T2-high signal.^5,12)^ In the present case, because most of the tumor comprised UES cells, these previously reported imaging features of UES could be observed.

Regarding the pathological features of UESL cells, no specifically differentiated cells, such as blood vessels, rhabdomyosarcoma, smooth muscle, fat, or nerves, are found. UESL shows characteristic proliferation of undifferentiated mesenchymal tumor cells, composed of stellate and spindle-shaped cells, with giant cells sometimes present in the mixture. Nuclear atypia is pronounced, nuclear fission is conspicuous, and Ki67 positivity typically exceeds 30%. Immunostaining typically yields positive results for the mesenchymal markers vimentin, α-1 antitrypsin, and CD68, while HepPar1, AFP, α-smooth muscle actin, myogenin, synaptophysin, and CD117 are often negative.^5,6)^ The results of immunostaining of the UESL component in the present case are shown in **Table 1** and were similar to those in previous reports, with positive staining for mesenchymal markers as well as α-1 antitrypsin. The tumor in the present case exhibited pathologically complex features in the adenocarcinoma component. While CK7 and CK20, markers of cholangiocarcinoma epithelium, showed focal positivity, CDX-2 and CK20, which are expressed in colon cancer, also showed positivity (**Fig. 4D**). Additionally, hepatocytes showed partial positivity, while the mesenchymal marker vimentin showed focal positivity; these findings were distinct from the typical findings of intrahepatic cholangiocarcinoma (**Table 1**). Therefore, although the adenocarcinoma component showed signs of bile duct differentiation, there were some atypical features that distinguished it from intrahepatic cholangiocarcinoma. In previous reports, normal hepatocytes and bile duct structures were found at the tumor margins (between the periphery of the lesion and the pseudocapsule) in UESL,^1)^ probably because of engulfment of the surrounding liver tissue during tumor development. In our case, adenocarcinoma rather than normal bile ducts was found at the periphery of the tumor, which differs from the characteristics of previous UESLs.

HCS is a liver tumor comprising a mixture of carcinoma and sarcoma.^13)^ The pathogenesis of HCS has been suggested to involve a variety of factors, including the collision of multiple stem cell-derived tumors or dedifferentiation of sarcoma components from a monoclonal epithelial tumor.^14)^ A common pathological feature is the presence of a transition zone between carcinoma and sarcoma within the tumor.^15,16)^ Bin et al. reported a review of 63 HCS cases, with hepatocellular carcinoma accounting for 60.3% of the epithelial tumor component, and osteosarcoma, undifferentiated pleomorphic sarcoma, and rhabdomyosarcoma accounting for most of the sarcoma component (20.6%, 14.3%, and 14.3%, respectively).^17)^ In our case, the sarcoma component consisted of UESL. However, Bin et al. did not include data on adenocarcinoma components. Therefore, cases of UESL with adenocarcinoma similar to the present case are extremely rare in the literature.

Several possible mechanisms may underlie the development of the tumor in the present case, including collisions between tumors of different origins (in our case, adenocarcinoma tissue may have been incorporated into sarcoma cells) and development of different tumors from the same stem cell, as well as the HCS carcinogenesis process.^18)^ In addition, the “bystander effect,” a phenomenon in which tumor cells induce cancerous transformation of normal adjacent cells, has previously been reported in the literature.^19)^ Our patient had a history of hepatitis C virus (HCV) infection and achieved a sustained virologic response with drug therapy. Zhou et al. conducted a meta-analysis and reported that HCV infection increases the risk of cholangiocarcinoma.^20)^ Additionally, Navas et al. proposed the hypothesis that HCV proteins are involved in cholangitis, liver fibrosis, and carcinogenesis, based on the analysis of cholangiocarcinoma samples with HCV infection.^21)^ Accordingly, our patient’s history of HCV infection may have been a contributing factor to carcinogenesis in the adenocarcinoma component. Although the clinical and pathological data obtained from the present case alone did not allow us to determine the mechanism of development of this rare tumor, we believe that the clinical findings will be valuable in elucidating the pathology of UESL or HCS.

## CONCLUSIONS

We presented a unique case of UESL containing UES cells and adenocarcinoma cells at the periphery. A combination of UESL and adenocarcinoma is extremely rare, even among HCSs. The findings from this case provide valuable insights into the pathological features of UESL.

## ACKNOWLEDGMENTS

None.

## DECLARATIONS

### Funding

No grant support or funding was received from public institutions or private enterprises.

### Authors’ contributions

AW reported this case and wrote the manuscript.

YE, MSu, TT, TY, JI, and MSo treated the patient and helped draft the manuscript.

MI assisted in diagnosing the pathological findings.

KS and HO critically revised the manuscript.

All the authors have read and approved the final manuscript.

### Availability of data and materials

The dataset supporting the conclusions of this article is included within the article.

### Ethics approval and consent to participate

This work does not require ethical considerations or approval.

### Consent for publication

Informed consent was obtained from the patient for the publication of this case report and accompanying images.

### Competing interests

The authors declare that they have no competing interests.
